# OA12.03. Integrative medicine at academic health centers: a survey of clinicians' educational backgrounds and practices

**DOI:** 10.1186/1472-6882-12-S1-O47

**Published:** 2012-06-12

**Authors:** G Ehrlich, T Callender, B Gaster

**Affiliations:** 1 University of Washington, Seattle, USA

## Purpose

Integrative medicine is a relatively new field which seeks to combine a wide range of conventional and nonconventional approaches to patient care. Many academic health centers have now established integrative medicine clinics, yet little is known about the clinicians who practice at them.

## Methods

We conducted a nationwide survey of clinicians (MD's, DO's, PA's, and nurse practitioners) who practice at integrative medicine clinics which are affiliated with academic health centers, seeking to characterize their educational backgrounds, clinical practices, and participation in research and education activities.

## Results

We received completed surveys from 136 of 162 clinicians (84% response rate). The integrative therapies that clinicians most often reported providing themselves were breathing exercises (66%), herbal medicine prescribing (61%), meditation (44%), and functional medicine (34%). The integrative therapies that clinicians most often referred their patients for were acupuncture (96%), massage (92%), yoga (85%), and meditation (79%). Respondents reported spending a mean of 20% of their time training medical students, and 63% had participated in research in the past year.

## Conclusion

This survey provides the first national assessment of clinicians practicing integrative medicine at academic health centers. These clinicians employ a wide variety of complementary and alternative therapies and appear involved in the research and education missions of their academic health centers.

